# Computational Studies of the Photodegradation Mechanism of the Highly Phototoxic Agent Benoxaprofen

**DOI:** 10.1021/acsomega.2c03118

**Published:** 2022-08-11

**Authors:** Klefah A. K. Musa, Leif A. Eriksson

**Affiliations:** † Department of Medicinal Chemistry, Pharmacy College, El-Mergib University, Al-Khoms 18342, Libya; ‡ Department of Chemistry and Molecular Biology, University of Gothenburg, 405 30 Göteborg, Sweden

## Abstract

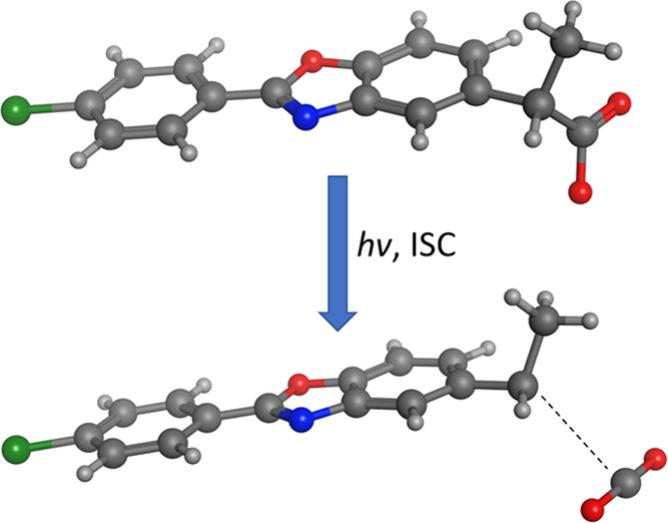

Computational quantum chemistry within the density functional theory (DFT) and time-dependent density functional theory (TD-DFT) framework is used to investigate the photodegradation mechanism as well as the photochemical and photophysical properties of benoxaprofen (BP), a non steroid anti-inflammatory molecule (2-[2-(4-chlorophenyl)-1,3-benzoxazol-5-yl] propanoic acid). BP is a highly phototoxic agent that causes cutaneous phototoxicity shortly after its administration. On the grounds of concern about serious side effects, especially hepatotoxicity, it was withdrawn from the world market after only 2 years of its release. Our study shows that the drug has the capability to absorb radiation in the UV region, mainly between 300 and 340 nm, and undergoes spontaneous photoinduced decarboxylation from the triplet state. It shows very similar photochemical properties to the highly photolabile non-steroidal anti-inflammatory drugs (NSAIDs) ketoprofen, suprofen, and tiaprofenic acid. Like ketoprofen, BP can also decarboxylate from excited singlet states by overcoming low energy barriers. The differences in molecular orbital (highest occupied molecular orbital (HOMO) and lowest unoccupied molecular orbital (LUMO)) distributions between the neutral and deprotonated BP, their absorption spectra, and the energetics and fate of various photoproducts produced throughout the photodegradation are discussed. Initiation and termination of decarboxylated BP radical species and initiation of propagating lipid peroxidation reactions due to the addition of molecular oxygen giving rise to the corresponding peroxyl radical are also explored in detail.

## 1 Introduction

**Figure 1:**
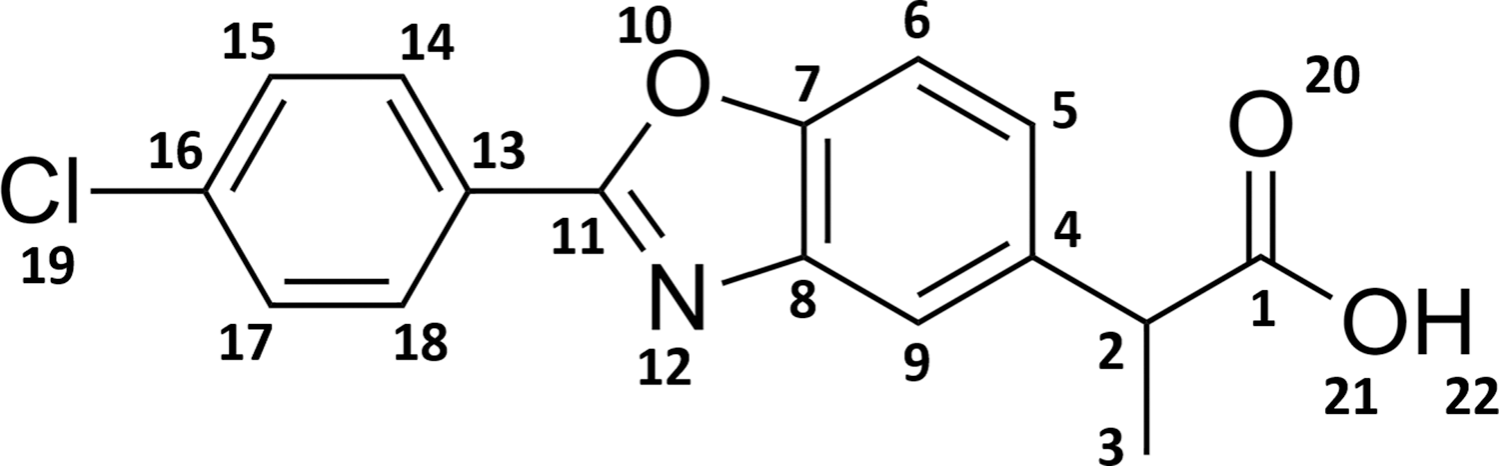
Chemical structure of benoxaprofen and atomic numbering used in the study.

Benoxaprofen (BP), 2-[2-(4-chlorophenyl)-1,3-benzoxazol-5-yl] propanoic acid, Figure 1, is a nonsteroidal anti-inflammatory drug (NSAID) introduced to clinical practice in 1980. It has a long plasma half-life, which means it only needs to be administered once daily. The drug was investigated in various comparative studies with similar NSAIDs to determine its utilization and pharmacokinetics in the treatment of, e.g., rheumatoid arthritis,^1^ osteoarthritis,^2^ and ankylosing spondylitis.^3^ BP is a weak inhibitor of prostaglandin synthetase and consequently has less gastric irritation effects than most NSAIDs. It efficiently inhibits the lipoxygenase enzyme responsible for converting arachidonic acid to potent mediators, such as hydroxyl derivatives and leukotrienes, which play important roles in inflammation and hypersensitivity reactions.^4,5^ Generally, BP exhibits notable anti-inflammatory, analgesic, and antipyretic activity in animals.^6^

Besides the therapeutic benefits, several off-target effects have also been noted during treatment with BP. In comparison with other NSAIDs, BP has a unique side-effect profile^7^ and shows fewer gastrointestinal side effects than other NSAIDs,^8−10^ with peptic ulceration reported in only 0.4% of patients^11^ along with rare cases of hemorrhage.^12^ However, from the early trials, BP was noted to generate an unusually large number of cases with increased photosensitive dermatitis and onycholysis.^13,14^ Photosensitivity occurred in half of the patients treated during summer, resulting in the withdrawal of BP in 30.2% of the patients experiencing this.^7^ Photosensitive dermatitis is a physical response to reactions occurring when the BP molecule is exposed to particular wavelengths of UV light and is neither an allergic nor immunological phenomenon.^15^ The intensity of sunlight, the BP concentration in the blood, and the degree of skin pigmentation are important factors related to rash formation.^16^ In addition, milia (milk spots) on the face were also reported during use of BP.^15−17^

The cutaneous phototoxicity was reported to occur shortly after administration.^18^ The photosensitivity mechanism of BP causing cell membrane damage was studied using red blood cells as a model system. The results obtained indicate that the photosensitivity mechanism for membrane disruption can be both oxygen-dependent and oxygen-independent. In the presence of oxygen, the photohemolysis is more rapid than in the absence. The mechanism was proposed to involve initial photodecarboxylation of BP, giving a lipophilic photoproduct that subsequently initiated lipid peroxidation and membrane damage.^19^ In an earlier study, BP showed to give rapid cell lysis of human erythrocytes once these were irradiated in the presence of oxygen. The lysis began after 10 min and was completed within half an hour from the exposure. The photohemolysis was also noted under anaerobic conditions; in this case, the onset was delayed for more than 20 min and the complete lysis occurred after ∼100 min. The main photoproduct of BP, 2-(4-chlorophenyl)-5-ethylbenzoxazole, was almost as effective as BP itself in causing photohemolysis.^20^

BP undergoes both type I and type II reactions. In aerated solutions (in the presence of oxygen), singlet oxygen, superoxide, and radicals were detected, whereas irradiation of anaerobic solutions of BP in ethanol resulted in hydrogen abstraction from the solvent to yield hydroxyl and ethoxyl radicals.^21^ BP has also been shown to enhance DNA cleavage in buffered aqueous solution (pH 7.4) upon irradiation at 300 nm. In the deaerated solutions, the number of single-strand breaks is 3 times higher in the presence of BP than in its absence. However, in aerated solutions, the rate of DNA cleavage is decreased. In addition, the mechanism of BP-induced DNA cleavage shows that the photoactive agent is the decarboxylated species and that the photodecarboxylation of BP is much faster than the photo-cleavage of DNA.^22^

The photosensitizing efficacy of the drug *in vivo* is, to some extent, correlated to the absorption spectrum of the drug *in vitro.*(23) BP provokes phototoxicity reactions in humans where the exposure to either solar simulating radiation or a broad UV-A wavelength band to the skin of BP-treated subjects produces intense itching and burning sensations, followed by the development of classical wheal and flare responses within 2–4 min. In addition, urticaria is provoked when using radiation with wavelengths between 320 and 340 nm. The phototoxic urticaria provoked by BP is suggested to be caused by a direct photolytic effect on human dermal mast cells.^28^ BP and its main decarboxylated photoproduct show similar absorption patterns, where for the former, the maximum absorption peak is found at 305 nm with ϵ = 3.0 × 10^4^ M^–1^ cm^–1^, and for the latter, λ_max_ = 307 nm and ϵ = 2.8 × 10^4^ M^–1^ cm^–1^. However, BP is different from its main photoproduct in that the latter is not water-soluble due to the absence of a carboxylic acid group that is deprotonated at physiological pH.^19^

BP was only available in the market for around 2 years, after which it was withdrawn on the grounds of its serious side effects. A total of 61 deaths were reported among patients treated with this drug, in addition to 3500 reports on adverse reactions received by the Committee on Safety of Medicine.^16^ Hepatotoxicity was the main serious side effect of BP leading to its withdrawal from the world market.^24^

Although BP was withdrawn from the market due to its fatal liver toxicity,^25−27^ the severe photosensitivity reactions caused in comparison with other NSAIDs make it a useful model in the study of photodegradation mechanism of highly phototoxic drugs and compared with the photodegradation pathways of other NSAIDs. This may in turn help in the prevention of phototoxic side effects of newly designed compounds or to understand and improve the properties of existing ones.

**Scheme 1:**
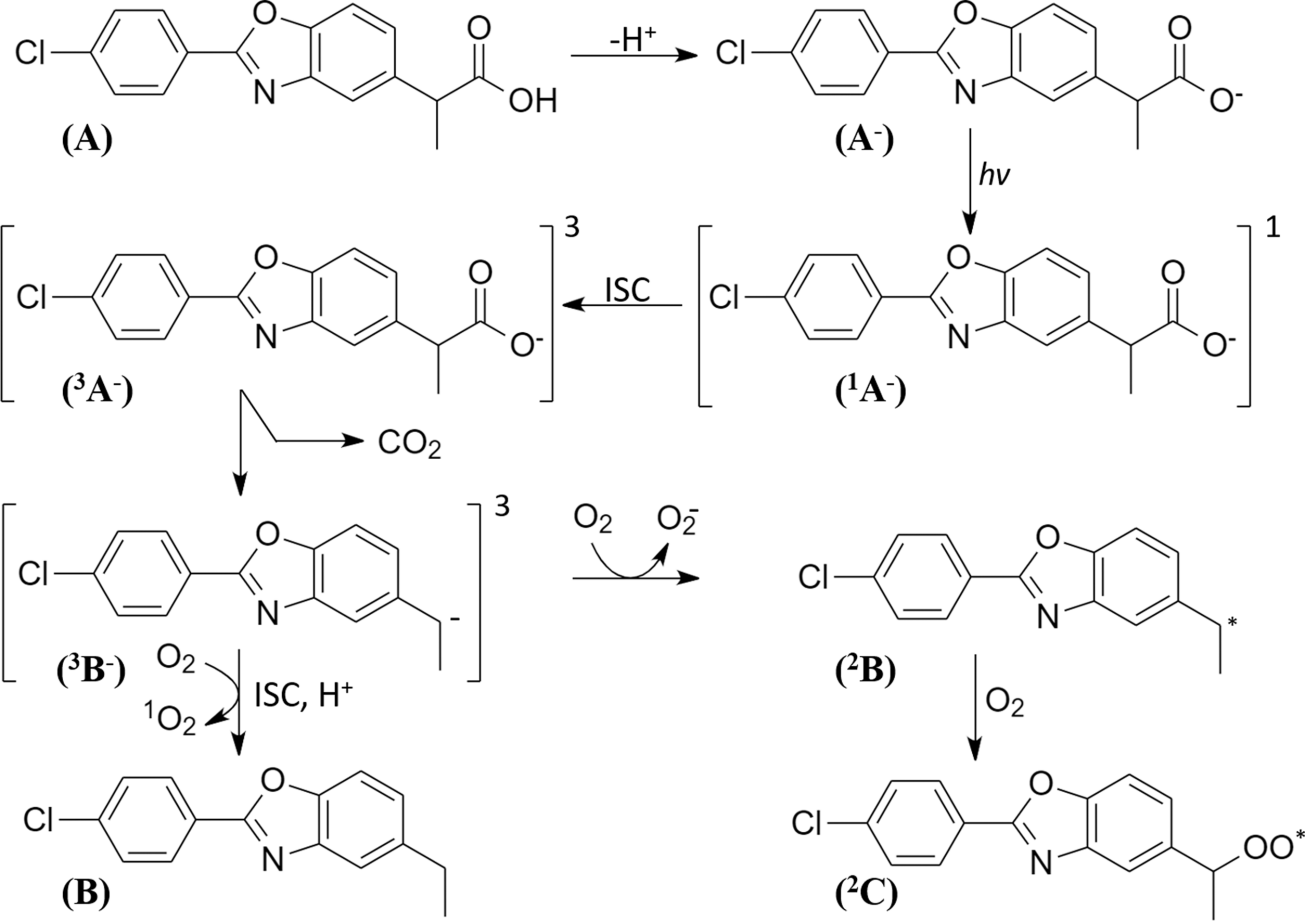
Proposed Photodegradation Mechanism of BP

Based on our previous studies of similar NSAIDs^29−33^ and available experimental data for BP, the proposed photodegradation mechanism is shown in Scheme 1. Because the compound has a low p*K*_a_ value of 3.5,^34^ it will predominately be in its deprotonated form under physiological pH. The deprotonated species has the capability to absorb UV light and thus reach an excited singlet state whereby, via intersystem crossing (ISC), a triplet state formed. Decarboxylation is expected to occur either from an excited singlet state and/or a triplet state, forming the decarboxylated species. The latter species has several pathways for termination, e.g., reaction with molecular oxygen forming singlet oxygen or peroxide radical derivatives. All of the pathways presented in Scheme 1 will be discussed in detail in this study.

## 2 Methodology

The HF-DFT framework in the form of the hybrid functional B3LYP^35−37^ in conjunction with middle size basis set 6-31G(d,p) was used to obtain the structures of all compounds described in Scheme 1 using the GAUSSIAN 03 package.^38^ At the optimized geometries of all species studied, zero-point vibrational energies (ZPE) were evaluated, as were free energy corrections at *T* = 298 K.

**Figure 2:**
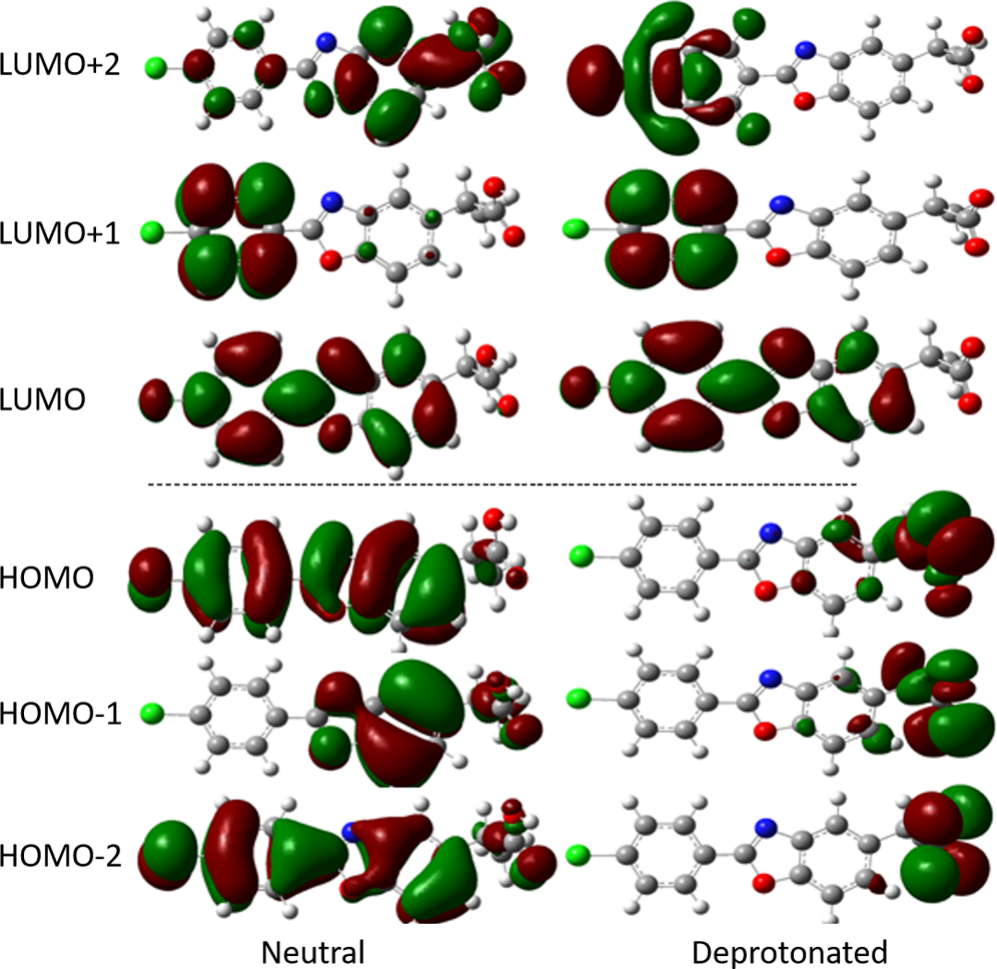
Molecular orbital distributions of the neutral and deprotonated forms of BP.

To describe the effect of the surrounding medium, single-point calculations were performed at the same level of theory using the integral equation formalism of the polarized continuum model (IEF-PCM) of Tomasi and co-worker.^39−41^ Bulk water was included as a solvent with a value of 78.9 for the dielectric constant ε.

At the same level of theory, electron and proton affinities, ionization potentials, and excitation energies were obtained. Atomic charges and spin densities were extracted using Mulliken population analysis.

Time-dependent DFT (TD-DFT)^42−44^ calculations were performed for excitation energies and when exploring the possibility for the decarboxylation process to occur from different excited states. The excitation energies tend to be overestimated by ∼0.2 eV at this level of theory, leading to a slight blue- shift of the peaks in the computed spectra. In addition, under the methodology employed herein, solvent effects have very little influence on the absorptions and were hence not included in the TD-DFT calculations. The atomic numbering scheme used for the different compounds follows that of Figure 1.

In a previous study based on the same methodology, we performed test calculations using a range of basis sets (such as 6-31g(d,p) and 6-311g(d,p) with or without diffuse functions) on the similar molecular size drug diclofenac and its main photoproduct (8-chlorocarbazole).^45^ The effects on absorption spectra and transitions are within a few nanometers, and we have thus for computational reasons used the B3LYP-6-31G(d,p) level of theory throughout for the current BP photodegradation mechanism.

Although more advanced methodologies are currently available for the computation of absorption spectra, retaining a well-established (and in the present context very successful) methodology enables us to make comparisons to the photodegradation mechanisms of previously studied NSAIDs explored at the same level of theory.^29−33^

## 3 Results and Discussion

### 3.1 Redox Chemistry of Benoxaprofen

The optimized structures of neutral BP (**A**), its radical anion and radical cation (**A**^–*^ and **A**^+*^), as well as its deprotonated species (**A**^**–**^) are displayed in Figure S1 in the supporting information. The main difference in the optimized structures is a change in the C_1_–C_2_ bond length, which is relevant for decarboxylation, from 1.523 in the neutral form (Figure S1A), to 1.599 Å in the singlet ground state of the deprotonated species (Figure S1D). However, very small changes are seen in this bond compared to the neutral species for the radical anion and radical cation (Figure S1B,C, respectively). Instead, a notable shortening of the C_11_–C_13_ bond length that connects the two aromatic systems is observed. In addition, the C_16_–Cl_19_ bond length is decreased or increased by 0.037/0.007 Å in the radical anion or cation, respectively, compared to the protonated form.

In Table 1, we list the absolute and relative ZPE-corrected energies in both gas phase and bulk solvent, along with the dipole moments obtained in aqueous solution. The electron affinity (EA) and ionization potential (IP) of this drug, computed in gas phase, are −12.8 and 171.1 kcal/mol, respectively. Once bulk solvation is included through the IEFPCM method, the values change to −50.2 and 135.7 kcal/mol for EA and IP, respectively. The energy difference in the gas phase between the neutral and deprotonated species is computed to be 346.2 kcal/mol, whereas in an aqueous solution, this is reduced to 295.3 kcal/mol. The obtained results herein are highly similar to those obtained for related NSAIDs (e.g., ketoprofen, ibuprofen, flurbiprofen, naproxen, suprofen, and tiaprofenic acid) studied previously.^29−33^

**Table 1:** B3LYP/6-31G(d,p) Zero-Point Energy Corrected Electronic Energies in Gas Phase, and IEFPCM-B3LYP/6-31G(d,p) Gibbs Free Energies in Aqueous Solution^a^

system	*E*_(ZPE)_	Δ*E*_(ZPE)_	Δ*G*_aq_^298^	ΔΔ*G*_aq_^298^	μ_aq_
**A** (singlet)	–1357.355757	0	–1357.42389	0	0.99
**A**^–*^ (doublet)	–1357.37611	–12.77	–1357.503923	–50.22	7.66
**A**^+*^ (doublet)	–1357.083158	171.06	–1357.207686	135.67	1.76
^**3**^**A** (triplet)	–1357.263496	57.89	–1357.333234	56.89	4.37
**A**^**–**^ (singlet)	–1356.804117	346.16	–1356.953373	295.25	26.15
^**3**^**A**^**–**^ (triplet)	–1356.758465	374.81	–1356.883861	338.87	8.27
^**3**^**B**^**–**^ (triplet)	–1168.185632	0	–1168.301414	0	5.13
**B**^**–**^ (singlet)	–1168.191946	–3.96	–1168.306845	–3.41	13.37
**B** (singlet)	–1168.802352	–386.99	–1168.855139	–347.47	2.82
B^*^ (doublet)	–1168.167575	11.33	–1168.221462	50.17	2.98
^**2**^C^*^ (doublet)	–1318.512696		–1318.574412		2.91

a Absolute energies, relative energies and dipole moments are in au, kcal/mol and Debye, respectively. Labels of the different systems refer to Scheme 1.

Mulliken atomic charge distributions of all species studied in the proposed mechanism are shown in Table S1. The protonated parent compound and its radical anion, radical cation, and deprotonated forms have local charges on the carboxylic moieties (O_20_–C_1_–O_21_–(H_22_)) computed to be −0.045, −0.101, 0.029, and −0.684 e^–^, respectively. The computed dipole moments of these species (Table 1) show a change by ∼6.7 and 0.8 Debye between the parent drug compound and its radical anion and radical cation, respectively. The dipole moment for the deprotonated form increases by more than 25 Debye compared to the parent compound due to the highly localized negative charge, in agreement with the obtained Mulliken charge distribution. For the decarboxylated molecules (**B** and **C** of Scheme 1), the charges are localized mainly on O_10_, C_11_, and N_12_ in the heterocyclic ring (Table S1).

The unpaired electron densities of the various species are displayed in Table S2. The unpaired electron density is in the radical anion localized on atoms C_9_ and C_11_ of the bicyclic system and the alternating atoms C_14_, C_16,_ and C_18_ of the phenyl ring. In the radical cation, the unpaired electron density is localized on atoms C_5_, C_7,_ and C_8_ of the bicyclic system and C_16_ of phenyl moiety of BP. The main unpaired spin density of the decarboxylated molecules ^3^**B**^–^ and ^2^B^*^ is localized on C_2_ connected to the carboxylic moiety prior to decarboxylation. For the peroxyl radical species ^2^C^*^, the main unpaired spin density is localized on the −OO^*^ group which is added to C_2_ upon the reaction of ^2^B^*^ with molecular oxygen (Scheme 1).

The highest occupied molecular orbitals (HOMOs) and lowest unoccupied molecular orbitals (LUMOs) of the protonated and deprotonated forms of BP were computed to provide a setting for the photochemistry of the drug (Figure 2). The HOMO and HOMO–2 of the neutral form are delocalized throughout the aromatic systems, whereas HOMO–1 of the same species is localized mainly on the benzoxazole moiety. In contrast to the neutral form, the HOMO, HOMO–1, and HOMO–2 of the deprotonated species are localized entirely on the propanoic acid group of BP. The LUMO, LUMO+1, and LUMO+2 of the protonated species are localized on the aromatic systems, the peripheral aromatic benzene ring, and aromatic benzoxazole ring (and to a lesser extent on the propanoic group), respectively. For the deprotonated form of BP, the LUMO, and LUMO+1 are essentially identical to those of the neutral species, whereas LUMO+2 is localized on the aromatic benzene moiety and the chlorine substituent. The large differences in the distribution of the occupied molecular orbitals between the neutral and deprotonated species of BP are reflected in the Mulliken atomic charge distribution on the carboxylic group, which in the deprotonated form is −0.684 , but only −0.045 in the protonated form of BP. This will in turn also have a considerable impact on the photochemical behavior of the protonated versus the deprotonated form.

### 3.2 Excitation of Benoxaprofen and Its Deprotonated Form

**Figure 3:**
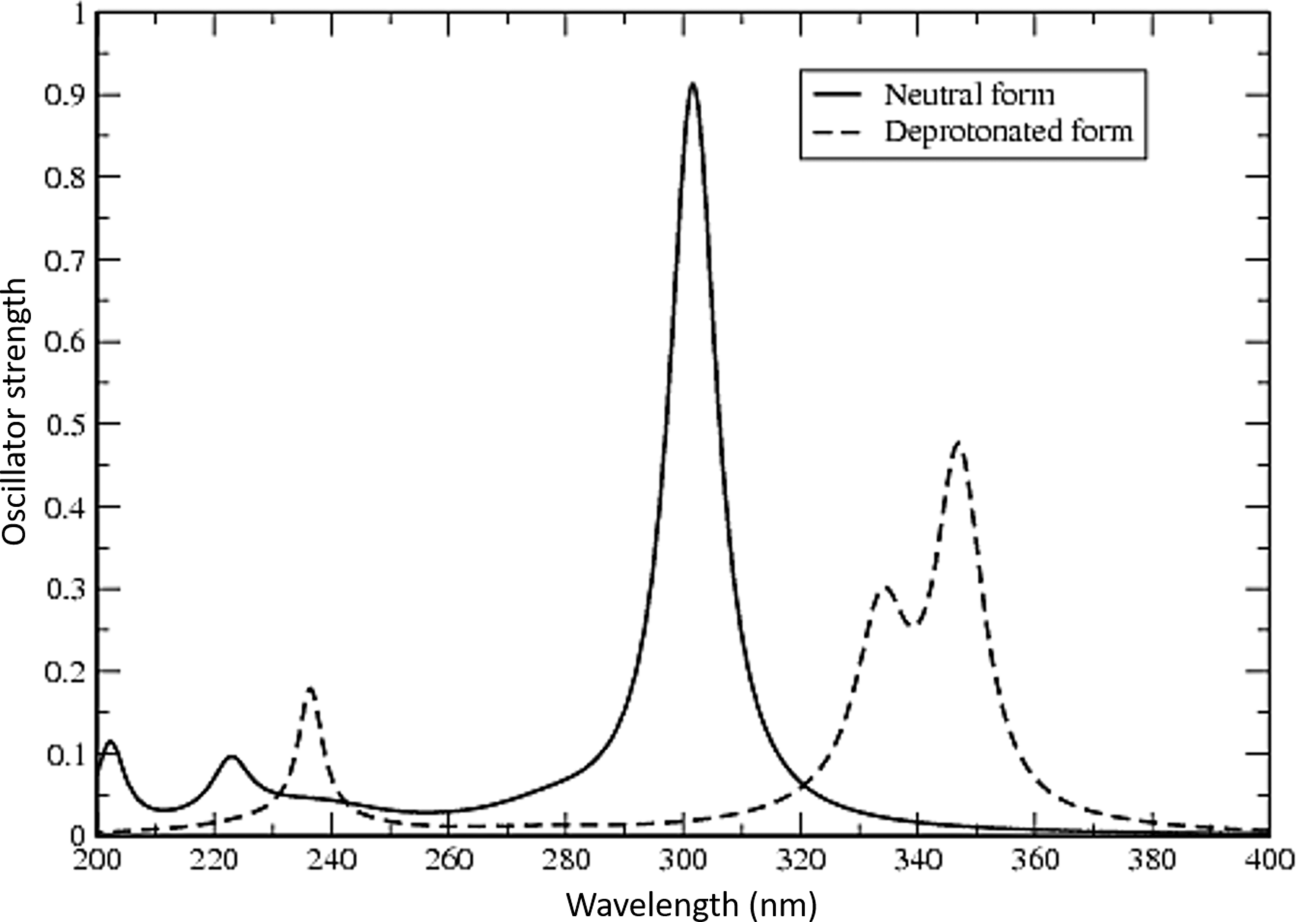
Computed absorption spectra of BP in its neutral (solid line) and deprotonated (dashed line) forms, obtained at the TD-B3LYP/6-31G(d,p) level.

The initial photodegradation step of BP, similar to previously studied NSAIDs, is the excitation of **A** in its protonated or deprotonated form to one of the first excited singlet states followed by intersystem crossing (ISC) to the first triplet state. Because of the low p*K*_a_ value of BP (3.5^34^) and the structural changes upon deprotonation mentioned above, photodegradation is more likely to occur from the **A**^**–**^ form. The computed UV absorption spectra of **A** and **A**^**–**^ are displayed in Figure 3. The two spectra are markedly different, which also relates to the differences in MO distribution, Figure 2.

For the neutral form, the first vertical excitation from HOMO to LUMO occurs at λ = 301 nm (∼95 kcal/mol), in the UV region of the spectrum, with an oscillator strength of 0.912. This indicates a high probability and forms the main absorption peak of this species. Two more absorption peaks are found at λ = 222 and 202 nm, with oscillator strengths equal to 0.101 and 0.139, respectively.

This group of NSAIDs has been observed experimentally to exhibit negligible absorptions in the visible region of the spectrum,^23^ a phenomenon also observed for the deprotonated NSAIDs in our previous computational studies,^29−33^ and is related to the extensive charge transfer nature of the transitions. The same is also found for deprotonated BP (cf. Figure 2); due to their negligible (zero) probabilities, these absorptions are, however, not photochemically relevant. The first observable absorption peak of this species is found at λ = 347 nm with an oscillator strength about half of that obtained for the neutral form, *f* = 0.477. This main absorption peak has a clear shoulder at a shorter wavelength, around λ = 333 nm, with an oscillator strength of 0.309. Another distinct absorption peak is found at wavelength 236 nm with an oscillator strength of 0.184. This is in line with experimental findings, according to which the photosensitivity of several patients was confined to wavelengths less than 340 nm. Irradiation between 310 and 330 nm was the most effective for producing a transient reddening of the skin in combination with severe burning, itching, and occasional wheals formation, whereas between 340 and 400 nm, irradiation produced immediate erythema localized to the site of exposure.^15^ It should also be noted that the method employed herein normally gives a blueshift of predicted absorptions (i.e., predicted wavelengths are shorter) by ∼0.2 eV. In the 300–400 nm interval, this corresponds to a shift of 10–20 nm.

Since the BP is an arylpropanoic acid derivative, photodegradation is expected to occur primarily from the deprotonated species at physiological pH, with photodecarboxylation as the dominant initial pathway, and radical formation and generation of reactive oxygen species to be expected via the photodegradation mechanisms outlined in Scheme 1.

Once the molecule is excited, the excited singlet state will upon ISC lead to the formation of the first triplet state. The optimized triplet state of the deprotonated species (^**3**^**A**^**–**^) lies 28.7 kcal/mol above the optimized ground state (**A**^**–**^); for the neutral form, the corresponding free energy difference is 57.9 kcal/mol (Table 1). Inclusion of bulk solvation has only a minor effect on these energies. The triplet energies of the optimized deprotonated and neutral species agree well with the vertical T_1_ energies computed from TD-DFT calculations of the deprotonated and protonated forms (28.7 and 64.6 kcal/mol, respectively). The optimized structures of the triplet states of the protonated and deprotonated species are displayed in Figure S1. For the neutral form, very small changes in geometry are observed, compared to the singlet ground state. In contrast, the deprotonated species undergoes spontaneous decarboxylation, which leads to decarboxylated BP (^**3**^**B**^**–**^) and carbon dioxide formation (Figure S1). This phenomenon is identical to what was observed for NSAIDs ketoprofen, suprofen, and tiaprofenic acid, where all of these drugs decarboxylated spontaneously without energy barriers from their corresponding deprotonated triplet states.^29,33^ For other NSAIDs such as ibuprofen,^30^ flurbiprofen,^31^ 6-methoxy-2-naphthylacetic acid (MNAA; the active form of nabumetone), and naproxen,^32^ we observed an elongation of the bond connecting the CO_2_ unit, as well as low energy barriers of 0.3, 0.4, 2.8, and 0.9 kcal/mol, respectively, for the decarboxylation to occur.^30−32^

The two first peaks of the absorption spectrum (Figure 3), at λ = 347 and 333 nm, respectively, correspond to excitation energies of 82.4 and 85.9 kcal/mol, respectively. For the protonated system, the initial (S_0_ → S_1_) excitation requires 95.0 kcal/mol, considerably higher than for the deprotonated system, and corresponds to the sharp absorption peak at 301 nm (Figure 3). The relaxed triplet states are formed via ISC; the neutral triplet requires 288.3 kcal/mol to deprotonate, which is slightly less than that required for singlet ground state deprotonation (295.3 kcal/mol). We can thus conclude that if the neutral form is excited, the p*K*_a_ of the triplet state is similar to that of the ground state, and will result in ^**3**^**A**^**–**^ formation. This scenario is similar to what we observed in our previous studies with other NSAIDs such as ketoprofen and ibuprofen.^29,30^

As mentioned above, the decarboxylation occurs spontaneously and without any energy barrier from the deprotonated species in the triplet state. To investigate if this process could also occur from an excited singlet state of the deprotonated species, the C_1_–C_2_ bond was scanned outward from the optimized value (1.599 Å) in steps of 0.1 Å. The structures were re-optimized at each new point, and the vertical excitation energies were calculated. In Figure S2, we show the resulting energy curves, including several of the lowest excited singlet states obtained at the TD-B3LYP/6-31G(d,p) level. The S_0_ ground state and the singlet state surfaces S_2_, S_3_, S_5_, S_6_, and S_8_ are strictly endothermic through the scan of the C_1_–C_2_ bond and show no sign of decarboxylation. However, the excited singlet state surfaces S_1_, S_4_, S_7_, and S_9_ show clear possibilities for decarboxylation by overcoming relatively low energy barriers; of these, S_7_ and S_9_ states correspond to the two peaks observed at 347 nm (82.4 kcal/mol) and 333 nm (85.9 kcal/mol) in Figure 3, respectively. As discussed above, the remaining absorptions have zero probability. In this context, the photophysical reactivity is very similar to the photodecarboxylation process of ketoprofen from its excited singlet states^29^ but differs from that of other NSAIDs such as ibuprofen and flurbiprofen.^30,31^

### 3.3 Fate of Decarboxylated BP Species

The decarboxylation process can occur either spontaneously without energy barrier from the triplet state of the deprotonated form, or from some of the excited singlet states by overcoming low energy barriers. The process generates carbon dioxide and the decarboxylated species. The latter may undergo several reactions, as depicted in the photodegradation mechanism (Scheme 1). These reactions can be summarized as follows
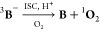
1
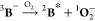
2
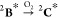
3

In the first reaction (1), the triplet decarboxylated species (^**3**^**B**^**–**^) reacts via ISC, protonation, and reaction with molecular oxygen to generate singlet oxygen. Through the protonation from the surrounding, the neutral singlet state of the decarboxylated species, **B**, is thus formed. The protonation energy of ^**3**^**B**^**–**^ forming the neutral singlet state **B** involves an energy gain of ∼387 kcal/mol in the gas phase, while in an aqueous solution, this is reduced to 347.5 kcal/mol. The relaxed singlet form, **B**^**–**^, lies ∼4 and 3.4 kcal/mol, below the triplet in the gas phase and aqueous solution, respectively, and may also readily become protonated in polar medium, given the estimated energy of solvated protons (268.7 kcal/mol).^48^ In this reaction, singlet oxygen is expected to be formed, since the energy required to form the singlet oxygen from molecular oxygen is previously estimated to be ∼22.5 kcal/mol.^46^

**Figure 4:**
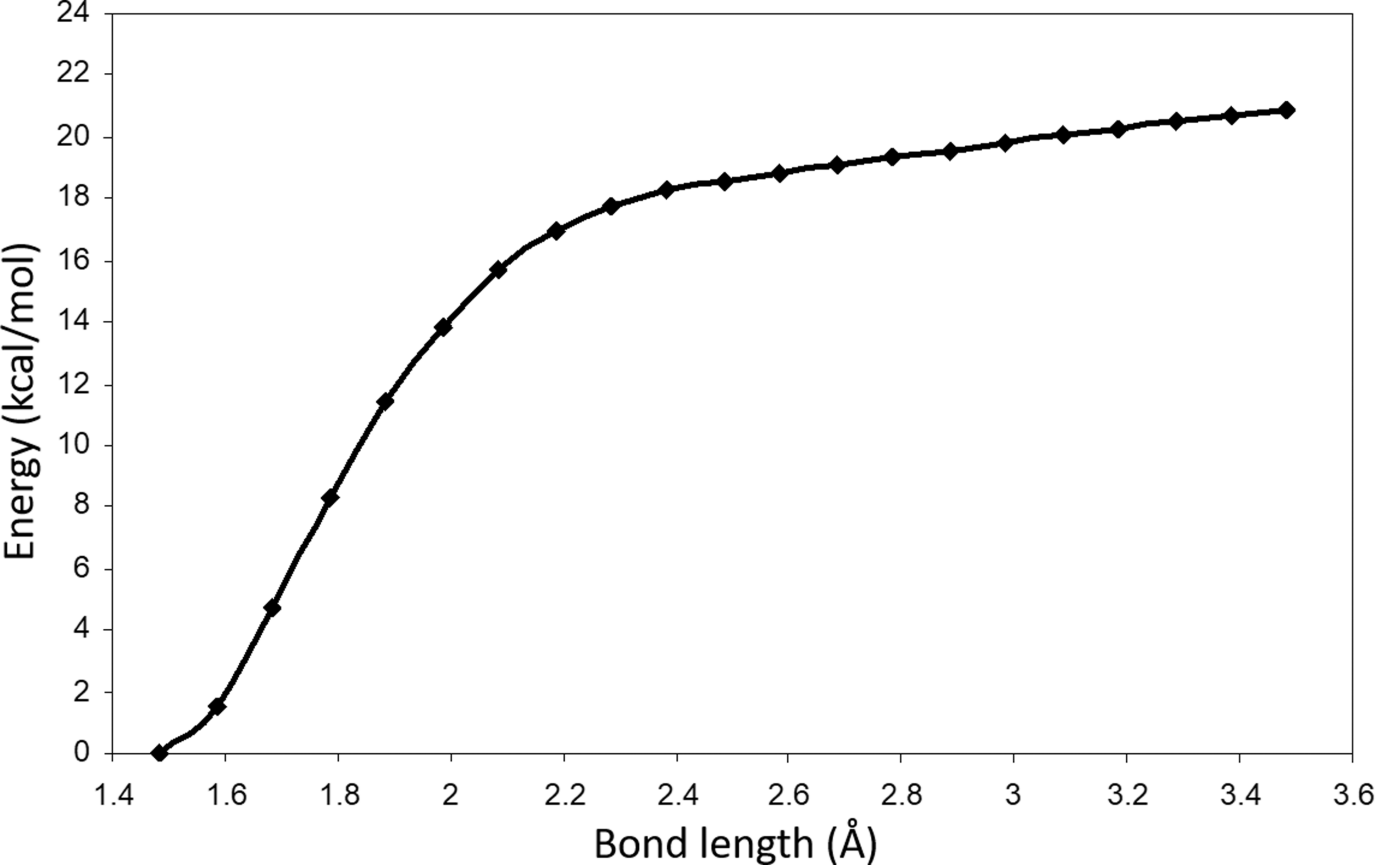
Reaction path for the formation of peroxyl radical ^**2**^C^*^ from ^**2**^B^*^ and molecular oxygen.

Another pathway of ^**3**^**B**^**–**^ is reaction 2, in which the doublet state of the decarboxylated species (^**2**^B^*^) is formed, via electron transfer to molecular oxygen. In vacuum, the ionization energy required to form ^**2**^B^*^ from the triplet state anion (^**3**^**B**^**–**^) is estimated to be 11.3 kcal/mol, while charge stabilization raises this to 50.2 kcal/mol in aqueous solution. This should be compared to the adiabatic electron affinity of O_2_, being ∼90 kcal/mol in aqueous solution.^47^ The formation of superoxide anions is hence a likely route. The formed doublet state (^**2**^B^*^) has a very reactive site at C_2_ with an unpaired spin density of 0.777 and will, by reaction with additional molecular oxygen lead to the formation of a peroxyl radical derivative (^**2**^C^*^), reaction 3. The addition of molecular oxygen to the decarboxylated species was explored by scanning the C_2_–OO distance in steps of 0.1 Å. In Figure 4, we display the energy diagram of this process. The energy difference between the peroxyl radical product (^**2**^C^*^) and the starting point with the reactants (molecular oxygen and ^**2**^**B**) separated by 3.5 Å, is 20.8 kcal/mol. This reaction is strictly exothermic with a clear change in slope at a C–O distance of about 2.1 Å. Reaction 4, which generates the peroxyl radical, is exergonic and will proceed spontaneously and without an energy barrier under aerobic conditions. These results are similar to those obtained for other NSAIDs studied at the same level of theory.^29,30^
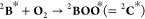
4
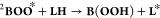
5
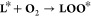
6
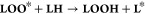
7

In addition to the reactions mentioned above, the peroxyl radical species ^**2**^BOO^*^ (=^**2**^C^*^) will also be an initiator of lipid peroxidation reactions. It can abstract a hydrogen atom from a lipid molecule forming a lipid radical species, which in turn adds molecular oxygen to yield a lipid peroxyl radical, LOO^*^, thus creating propagating radical damage. These processes are described in reactions 4–7. The propagation of radical damage cascade system (reactions 6 and 7) will begin and continue until terminated through, for example, by radical–radical addition or by the action of antioxidants such as vitamin E. Furthermore, the peroxyl radical species ^**2**^BOO^*^ (^**2**^C^*^) has the capability to react with other macromolecule systems such as DNA. As previously discussed, experimental data have shown the ability of BP to enhance DNA cleavage in buffered aqueous solutions (pH 7.4) upon irradiation at 300 nm, with the key reactive component being the decarboxylated form. In de-aerated solutions, the number of single-strand breaks is 3 times greater in the presence of BP than in its absence.^22^

## 4 Conclusions

BP is a highly phototoxic agent that causes cutaneous phototoxic reactions shortly after its administration. In the present study, the photochemistry of BP was investigated using DFT and TD-DFT methodologies to enable comparison with other NSAIDs previously investigated.^29−33^ Due to the low p*K*_a_ value of BP, the deprotonated species is predominant at physiological pH. The computed MO distributions show a clear difference between the neutral and deprotonated forms. For the neutral species, HOMO, HOMO-1, and HOMO-2 localize mainly in part of, or all of, the aromatic systems. For the deprotonated form, the HOMO, HOMO–1, and HOMO–2 are localized on the carboxylic moiety of the propanoic acid group. The absorption spectra of BP and its deprotonated form show that BP has the capability to efficiently absorb radiation in the UV region of the spectrum. For the neutral form, the first vertical excitation from HOMO to LUMO occurs at λ = 301 nm with an oscillator strength of 0.912. Two additional strong absorption peaks are found at λ = 222 and 202 nm, with oscillator strengths of 0.101 and 0.139, respectively. The main absorption peak of the deprotonated form is found at λ = 347 nm with an oscillator strength of 0.477. The findings are in good accordance with experimental observations.

From the computed results, we can conclude that the photodegradation of BP occurs via an initial decarboxylation process, which can occur either spontaneously from the triplet state of the deprotonated form or from a few excited singlet states by passing low energy barriers. The net outcome of this process is the generation of a decarboxylated triplet species. This carries a high unpaired spin density (>0.7 e^–^) at C_2_, the site of decarboxylation, which makes it very reactive before final termination: (i) The triplet decarboxylated species (^**3**^**B**^**–**^) can form the neutral singlet state of the decarboxylated species (**B**) and reactive singlet oxygen via ISC, protonation, and reaction with molecular oxygen, (ii) the doublet state of the decarboxylated species (^**2**^B^*^) can form via electron transfer to molecular oxygen, resulting in superoxide anions. The ionization energy of ^**3**^**B**^**–**^ in aqueous solution is estimated to be 50.2 kcal/mol, compared to the EA of O_2_ (aq), which is 90 kcal/mol. (iii) the addition of molecular oxygen to the formed ^**2**^B^*^ radical species. This is a strictly exothermic reaction which, under aerobic conditions, will be spontaneous and without energy barrier. (iv) The addition of molecular oxygen to the radical species can in turn initiate propagating lipid peroxidation and/or DNA cleavage. This process is terminated upon, e.g., radical–radical addition or by the action of antioxidants such as vitamin E.

## Supporting Information Available

Tables of Mulliken charge populations and spin densities for the different systems and figures showing key structural parameters for optimized structures and evolution of singlet excited states as a function of C–C distance during decarboxylation of ^**1**^**A**^**–**^ (PDF)

## Notes

Output files with optimized structures and energetics are available from the authors upon request.
