# Supplementary material for: Computational Studies of the Photodegradation Mechanism of the Highly Phototoxic Agent Benoxaprofen
Source: ACS Omega. 2022 Aug 11;7(33):29475–82. doi: 10.1021/acsomega.2c03118 (PMC9404164; doi:10.1021/acsomega.2c03118)
Supplement: Supplementary file 1 — ao2c03118_si_001.pdf [file ao2c03118_si_001.pdf]

Computational studies of the photodegradation mechanism of the highly phototoxic agent Benoxaprofen

Klefah A. K. Musa[a] and Leif A. Eriksson[b]

[a]Department of medicinal chemistry, Pharmacy college, El-mergib University, Al-khoms, Libya

[b]Department of chemistry and molecular biology, University of Gothenbury, 405 30 Göteborg, Sweden

---

Supporting Information

Table S1. Mulliken atomic charges p2

Table S2. Unpaired spin densities p2

Figure S1. Optimized structures and key geometric parameters p3

Figure S2. Energy curves for decarboxylation of $^{2}\mathbf{A}^{-}$ p4

Table S1. Mulliken atomic charges (B3LYP/6-31G(d,p) level) for selected atoms in BP and its photoproducts;cf Figure 2 of main text. For atomic labeling see Figure 1 of main text.

system	C1	C7	C8	O10	C11	N12	O20	O21	H22
**A**	0.589	0.322	0.235	-0.531	0.496	-0.545	-0.469	-0.487	0.322
**A^{-*}**	0.584	0.327	0.251	-0.567	0.419	-0.611	-0.498	-0.492	0.305
**A^{+*}**	0.600	0.360	0.270	-0.504	0.534	-0.508	-0.451	-0.465	0.345
**^{3}A**	0.589	0.361	0.270	-0.547	0.446	-0.546	-0.467	-0.485	0.324
**A^{-}**	0.540	0.304	0.232	-0.543	0.480	-0.554	-0.610	-0.614	
**$^{3}A^{-}$**	0.733	0.326	0.254	-0.568	0.417	-0.608	-0.374	-0.375	
**$^{3}B^{-}$**		0.324	0.254	-0.569	0.417	-0.610			
**B^{-}**		0.261	0.257	-0.544	0.462	-0.591			
**B**		0.317	0.237	-0.532	0.495	-0.545			
**$^{2}B^{*}$**		0.315	0.240	-0.532	0.496	-0.546			
**$^{2}C^{*}$**		0.236	0.324	-0.530	0.497	-0.544			

Table S2. Main components of the atomic spin densities (B3LYP/6-31G(d,p) level) for the radical species of BP and its photoproducts. For atomic labeling see Figure 1 of main text.

System	C2	C5	C7	C8	C9	C11	N12	C14	C16	C18
**A^{-*}**					0.137	0.198		0.162	0.277	0.159
**A^{+*}**		0.294	0.169	0.104					0.149	
**^{3}A**		0.353	0.194		0.239	0.297	0.334	0.268	0.433	0.279
**$^{3}A^{-}$**	0.738	0.321			0.337				0.276	
**$^{3}B^{-}$**	0.753	0.322	0.173		0.338	0.184			0.278	
**$^{2}B^{*}$**	0.777		0.179		0.269					
**$^{2}C^{*}$**	Spin densities of this species are located on the added –OO group; O (inner) and O (outer) have unpaired spins 0.300 and 0.694 e^{-}, respectively.									

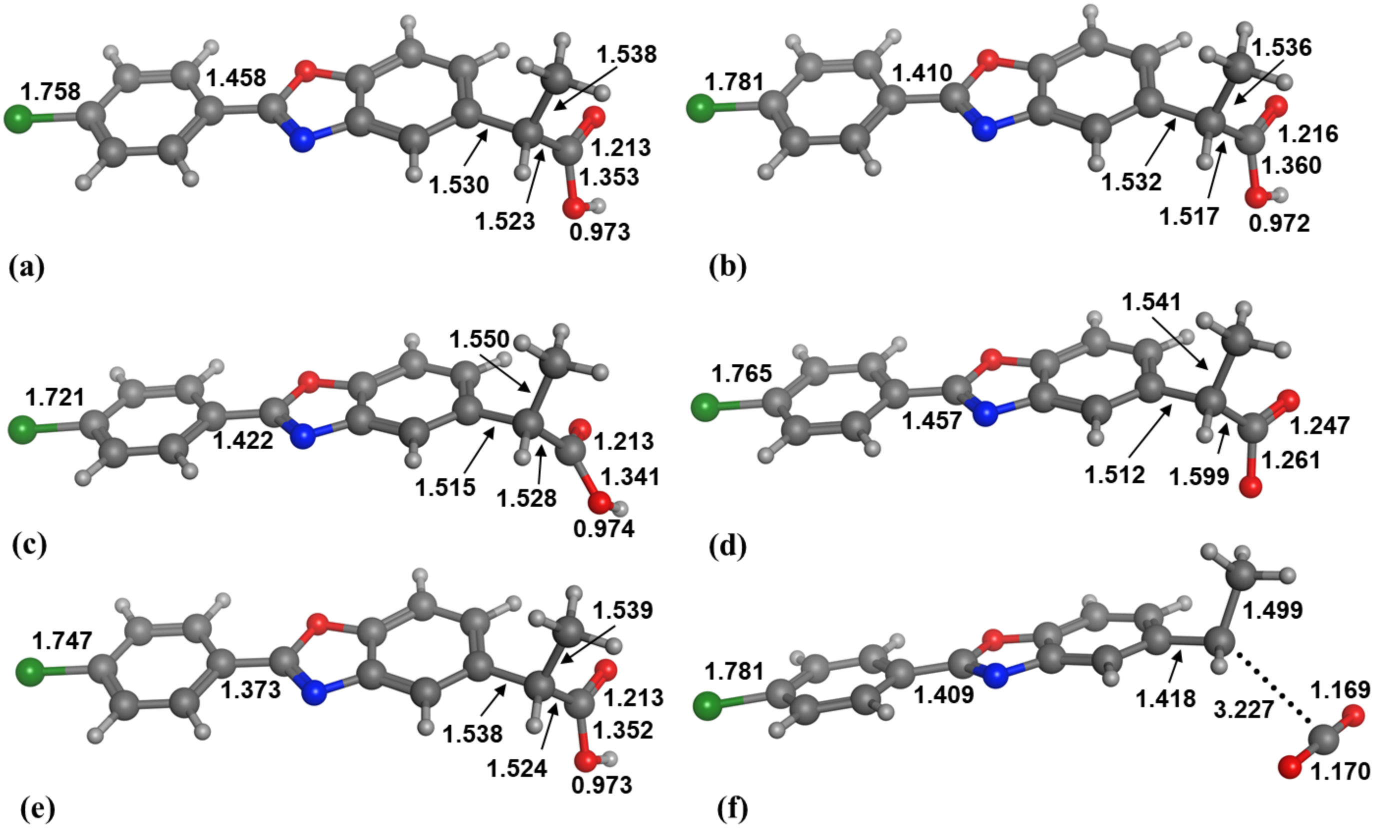

Figure S1. Optimized structures of BP. (a) Neutral ground state, **A**; (b) radical anion, $\mathbf{A}^{*-}$**;** (c) radical cation, $\mathbf{A}^{*+}$; (d) deprotonated ground state, $\mathbf{A}^{-}$; (e) triplet state of neutral species, $^{3}\mathbf{A}$; and (f) deprotonated triplet species, $^{3}\mathbf{A}^{-}$.

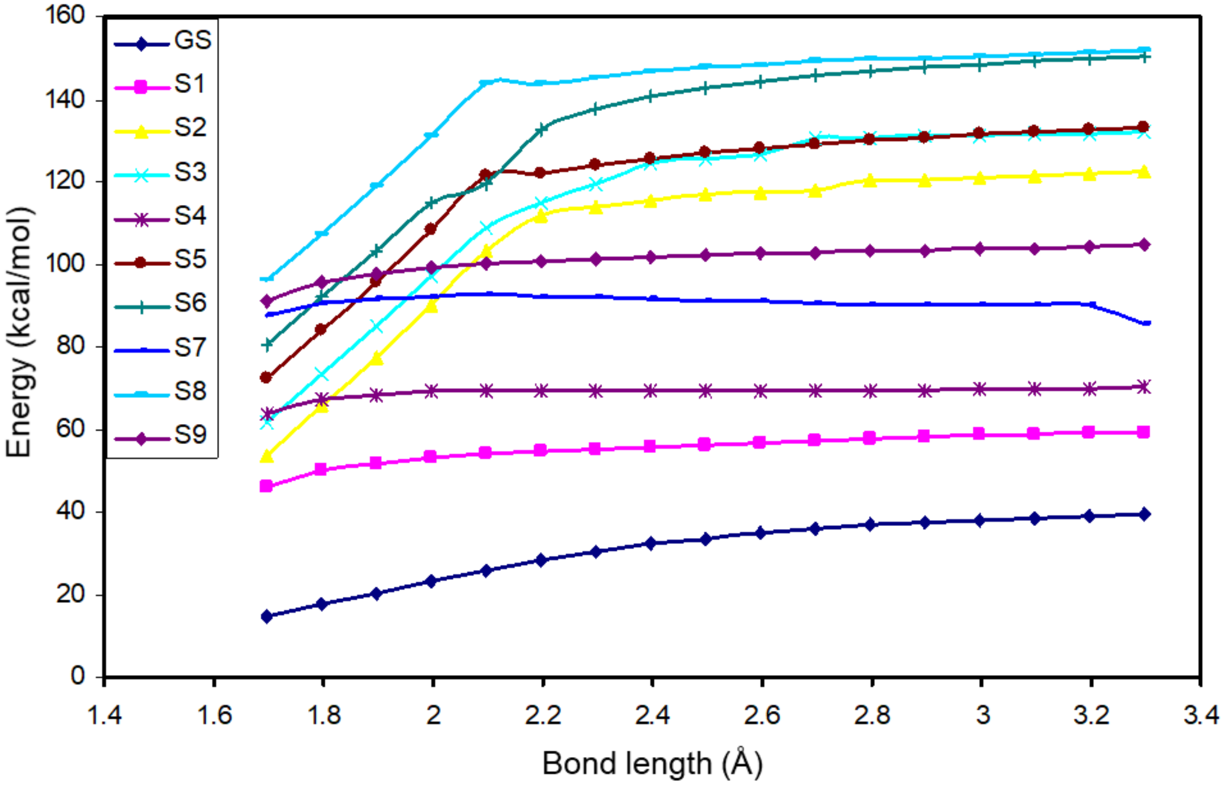

Figure S2. Energy curves for decarboxylation of BP from the deprotonated species $^{1}\mathbf{A}^{-}$. The ground state and the lowest excited singlet states are shown.
